# Knowledge and intentions regarding the Pap smear test among Saudi Arabian women

**DOI:** 10.1371/journal.pone.0253850

**Published:** 2021-06-24

**Authors:** Nawal A. Alissa

**Affiliations:** Department of Community Health Sciences, College of Applied Medical Sciences, King Saud University, Riyadh, Saudi Arabia; University of West London, UNITED KINGDOM

## Abstract

**Background:**

The knowledge of Pap smear and uptake of preventive behaviors to prevent cervical cancer are the most important contributors to the advanced stage of the disease. Knowledge is one of the most leading factors to predict the health behaviors and a helpful factor in performing screening procedures. This study aimed to investigate Saudi Arabian women knowledge of Pap testing in relation to their intention to undergo the test.

**Methods:**

An online survey including demographic characteristics (3 questions), knowledge (13 questions), and intentions (3 questions) towards Pap smear was completed by 467 Saudi Arabian women. Data were analyzed through SPSS version 22, using descriptive statistics and correlation to measure the relationship between knowledge, demographic factors, and intention.

**Results:**

The study found that average level of knowledge was 1.3 which is between high and moderate knowledge, and the average score for the intention was 2.88, to indicate that the intentions to uptake Pap smear among the participants were above the average. The study revealed a significant correlation between demographic factors and intention (p<0.01).

**Conclusion:**

Findings from the current study can inform health care providers about Saudi Arabian women knowledge of Pap smear and the intention to uptake the test. Strategies to motivate women to undergo Pap screening should be introduced.

## Introduction

Cervical cancer is the fourth most common cancer in women worldwide [1]. Globally, more than 570,000 women were diagnosed with cervical cancer and about 311,000 women died from the disease, a number that is expected to grow as the population age [1]. Cervical cancer is considered a major health problem in eastern, western, middle, and southern Africa [2]. In Saudi Arabia, cervical cancer incidence is low. In 2018, the number of cervical cancer cases is 316 and 158 deaths among Saudi Arabian women [3]. Cervical cancer is the 9th most frequent cancer among women in Saudi Arabia between the ages of 15 and 44 years. Although the incidence of cervical cancer in Saudi Arabia is lower than that of many other countries, it has increased significantly over the last two decades [4].

More than 40% of cervical cancer cases are diagnosed at advanced stages among Saudi Arabian women probably due to the lack of national screening programs in Saudi Arabia [5]. Cervical cancer is on the most preventable cancers [1]. Early screening and treatment of cervical cancer are important to decrease incidence and mortality. Early detection of cervical cancer can be obtained with Pap smear tests. Pap smear test is one of the effective methods to detect the cervical cancer [6]. The United States Preventive Services Task Force (USPSTF) recommends cervical cancer screening in women aged 21 to 65 years with Pap smear every 3 years, or for women age 30 to 65 years with a combination of Pap smear and HPV testing every 5 years [6]. The USPSTF recommends against screening with HPV testing alone or with Pap smear in women younger than age 30 years. It also recommends against screening in women younger than age 21 years and in women older than age 65 years who had adequate prior screening and not at high risk for cervical cancer [6]. The mortality rates for cervical cancer in developed countries have decreased dramatically in the past 25 years, due largely to cervical cancer screening using Pap tests, which allows for detection and treatment of precancerous lesions [7].

The knowledge of Pap smear and uptake of preventive behaviors to prevent cervical cancer are the most important contributors to the advanced stage of the disease. Knowledge is one of the most leading factors to predict the health behaviors and a helpful factor in performing screening procedures. However, knowledge alone is not sufficient since individual’s intention to uptake the preventive measure is also an important element in adopting preventive behaviors. Thus, the knowledge of Pap smear and intentions to undergo screening for cervical cancer are critical to primary prevention. In a study in United Arab Emirates 80% of the women had no knowledge of precancerous lesions [8]. In a similar study [9], examined the knowledge of Pap smear tests among Bahraini women in primary health care centers in Bahrain. The researchers found that of about 64% Bahraini women had never heard of a Pap smear procedure and only 40.7% had a Pap smear in their lifetime. Another study [10] found that Omani women knowledge about symptoms of cervical cancer and Pap smear was low at 38.7%, 35.3% and 7.6% among outpatients, staff, and students, respectively. Approximately half of the married outpatients had adequate overall knowledge as compared to none of the single women. Educational level was found to be significantly associated with outpatient knowledge, with the highest awareness levels among postgraduates and medical university graduates [10]. A research study among female health college students in Princess Nora University in Riyadh City, Saudi Arabia indicated that 95.7% of the students had a poor level of knowledge of cervical cancer [11]. Also, the study found that Pap smear was poorly recognized as a screening tool by most of students and misconception regarding primary and secondary preventive measures [11]. Generally, the studies in gulf countries [8–10]. and Saudi Arabia [11] revealed low knowledge about Pap smear test and there is a need for more education and promotion programs to increase the awareness of cervical cancer and Pap smear test in the population. Moreover, health education programs were recommended as an effective strategy in improving the level of knowledge on cervical cancer among the target population.

A research study examined the factors associated with the intention to undergo Pap testing, by level of sexual experience [12]. Findings of the study revealed that the subjective norm was the most important predictor of intention to undergo Pap testing [12].However, there is no previous studies about the knowledge of Pap smear testing in relation to the intention to undergo Pap testing among Saudi Arabian women. This information would be crucial in developing interventions to educate Saudi Arabian women on cervical cancer prevention measures. The results of this study will support greater awareness and prevention of cervical cancer and create targeted areas for future health promotion and education efforts. Findings of the study will be helpful to use knowledge of Pap smear testing in the prediction of Saudi Arabian women prevent behavior in relation to undergo Pap testing.

The continuous rise in cervical cancer around the world necessitates health professionals to pay more attention to this prevented disease. Health care providers could be a great contributor in reducing cervical cancer rates by participating in research on this important issue and by providing effective preventive measures. Even though cervical cancer morbidity and mortality rates are increasing among Saudi Arabian women, understanding and awareness of the disease are low [11].This lack of knowledge and awareness combined with the low uptake of preventive measures could put the women of Saudi Arabia at risk of having cervical cancer. These disturbing facts and statistics regarding the cervical cancer among Saudi Arabian women justify the need for the implementation of effective initiatives to prevent the disease.

This study may have an important impact on the focus of cervical cancer research in Saudi Arabia. Emphasis should be given to implementing interventions aimed at increasing awareness of cervical cancer in relation to undergo screening for cervical cancer to encourage healthy preventive behaviors among Saudi Arabian women, thereby reducing the risk of cervical cancer. It is the goal of this study to examine the knowledge of Pap testing in relation to the intentions to undergo the test and make recommendations for educational programs to increase the knowledge of cervical cancer preventive measure in relation to intentions to uptake Pap testing to decrease the incidence of cervical cancer among the target population.

## Methods

### Samples

The study participants consisted of a convenience sample of 467 Saudi Arabian women ages 18 years and above and from Riyadh, Saudi Arabia. Saudi Arabian adult women were recruited through online.

### Eligibility criteria

Eligible participants in this study included Saudi Arabian women. Additional inclusion criteria were age of 18 years and older and able to read Arabic and lived in Riyadh. Individuals of interest who did not meet all four criteria were excluded from the study.

### Instrumentation

The purpose of the study had proposed two constructs as its main target factors of study. The first construct was knowledge of Pap smear test. The second construct was intentions to having Pap smear test. Each construct was adapted from previous studies [13, 14].

The study was conducted by using a structured and online survey. This cross-sectional study was conducted through online recruitment of Saudi Arabian women aged 18 years and older in April 2018.

The survey was used as the research tool to measure participants’ knowledge of Pap smear test in relation to the intentions to undergo the test. The survey included information about demographic characteristics, about the knowledge of Pap smear, and the intentions of uptake Pap smear.

The survey for this study included 19 items. These items were organized into three sections. The first section of the survey consisted of demographic questions. They included the followings:

1. Age, item # 1: “*what is your age in years*?” Participants were provided a blank space to write in their specific age. Participants who listed an age below 18 were excluded from the study.
2. Marital Status, item #2: “*What is your marital status*?” Participants were given the following choices (1) Single, (2) Married, (3) Separated/Divorced, (4) Widowed, (5) Other. If participants chose other, they were provided a blank space to write in their specific marital status.
3. Educational Level, item #3: “*What is the highest degree or level of school you have completed*?” The selected options were (1) No schooling completed, (2) Some high school, no diploma, (3) High school graduate, diploma or the equivalent, (4) Some college credit, no degree, (5) Associate degree, (6) Bachelor’s degree, (7) Graduate degree.

The second section of the survey was Knowledge of the Pap Smear Scale to measure the first construct (knowledge of Pap smear test). The scale included thirteen items (from item #4 to item #16) was adapted [13] from Chamani, Alizadeh, and Kamalifard (2012). The item response options included yes and no responses. For example, participants were asked to answer yes or no with the statement:" Women should have Pap smears at least every three years*"*. To calculate knowledge score, correct answers were given score 1 and incorrect answers were given score 2. The total knowledge score was classified into three levels scores: 1 “High knowledge”, 1.5 "Medium Knowledge", and 2 "Low Knowledge". Higher composite scores on the overall scale indicated low knowledge of Pap smears.

The second construct (intentions to having Pap smears) was measured using the Behavioral Intention Scale. The Behavioral Intention Scale was the third section of the survey to measure behavioral intentions to having Pap smears with three items. The scale was adapted from [14]. Rise, Kovac, & Moan (2008). Item #17 asked the participants to report how likely or unlikely the following statement applies to them: “*In the coming three months*, *I intend to have Pap smears*.” To measure the intentions, a 5-point scale was used with very unlikely and very likely as endpoints. Item # 18 asked the respondents to report how often they intend to have Pap smears. Item # 19 asked the participants to indicate if they will plan to have Pap smears. The item response options included a 5-point Likert scale ranging from 1 (*likely*) to 5 (*unlikely*). Higher composite scores on the overall scale indicated decreased intention to adopt the preventive behavior.

All sections were combined to formulate the survey, the Knowledge and Intentions of the Pap Smear Survey. The three sections that were included in the current survey were the (1) Demographic Questions (3 items); (2) Knowledge of the Pap Smear Scale (13 items); and (3) Behavioural Intention Scale (3 items).

The instrument was translated from English to Arabic. The translation of the survey into Arabic was completed by an experienced translator, proficient in both Arabic and English.

### Data collection process

The survey was placed through the investigator’s Google web page and then distributed through a link to social networks (Twitter, Instagram, WhatsApp). The data were collected after the study was approved by the Institutional Review Boards (IRB’s) of King Saud University. Ethical approval for this study was obtained from the KSU Ethics Committee Review Board of the College of Applied Medical Science. Participants who met the inclusion criteria were asked to read the informed consent. Participants were notified that their participation in this study is voluntary, and they may decline answering questions or withdraw from the study at any time.

### Data analysis

The data were entered and stored in a computer database on the principal investigator’s computer. The data were checked for missing values and data entry errors. Statistical analysis was performed using SPSS Version 22. Descriptive statistical analysis was applied in the study with *p* < 0.05 considered significant. The independent variables in this study included knowledge of Pap smear and demographic characteristics. The dependent variable was the intentions to uptake Pap smear. This study analyzed the relationships between knowledge of Pap smear and the intentions to take the test. Descriptive statistics were obtained for all the variables studied. The Pearson Correlation Coefficient was applied to specify how two variables vary together, the knowledge of Pap smear, demographic characteristics, and intentions. Frequencies were to identify participants’ demographic characteristics including identification of age, educational level, and marital status. To measure the knowledge of Pap smear, composite scores on overall knowledge of Pap smear Scale were calculated. The mean of the composite scores was taken of the thirteen items to calculate a total knowledge score. The question response options included two options (yes and no). The questions were scored as *yes* = 1 and *no* = 2, with 1.5 as an average score. Higher numbers indicating lower levels of knowledge. To measure the intentions to have Pap smears, composite scores on the overall Behavioral Intention Scale were calculated. The mean of the composite scores was taken of the three items to calculate a total intentions score. The item response options included a 5-point Likert scale ranging from 1 (*likely*) to 5 (*unlikely*), with 2.5 as an average score. Higher composite scores on the overall scale, indicated decreased intention to take the test.

### Research design

A quantitative, exploratory, descriptive, correlational, and cross-sectional research design was used. A survey was used to collect data from the participants. The goal of using quantitative survey research is to determine the potential predictive power of the knowledge of Pap smears in having the test. The study is specifically a correlational design to determine whether the variables are correlated. The relationship between the independent variables, knowledge of Pap smear and demographic characteristics was measured using Knowledge of Pap Smear Scale and demographic factors. The dependent variable of intentions to have the test was examined using the Behavioral Intention Scale. All these instruments were combined to form the Knowledge and Intentions of the Pap Smear Survey. The instrument was administrated to through Google web page and then distributed through a link to social networks (Twitter, Instagram, WhatsApp).

## Results

A total of 467 women were included in the study. A total of 467 surveys were usable for data analysis. None of the survey were excluded because all the data was completed through an online survey that works by the mechanism of non- sending the questionnaire unless all answers are completed. Descriptive statistics, frequencies, and Pearson Correlation were used for statistical analyses. Ages ranged from 18 to 65 years. As shown in Table 1, majority of the participants (61.2%) were aged 18–29 years and a low percentage (7.5%) were aged 50–65 years. More than half of the participants (53.8%) were divorced at the time of survey, followed by married (42.8%), widowed (1.9%), and single (1.5%). Most of the study participants (83%) had received a bachelor’s degree.

**Table 1:** Frequency distribution by age category (n = 467).

Age Category	*N*	%
18–29	286	61.2
30–49	146	31.3
50–65	35	7.5
Total	467	100.0

The knowledge of pap smears was measured using the Knowledge of Pap Smear Scale. The participants were given the choices of yes and no to respond to thirteen items. Most participants 79.9% (n = 373) reported “yes” to answer the statement “Pap smears is most helpful way to detect pre-cancer and cancer of cervix”. Majority of the participants 62.9% (n = 294) knew that a Pap smear should not performed at both menstrual and non-menstrual period. Only 28% (n = 131) were aware that cervical cancer can be detected by Pap smear before manifestation of its symptoms, 32.5% (n = 152) knew that Pap smears can reduce deaths from cervical cancer and 42.2% (n = 197) knew that a Pap smear should be continued after menopause. The average score of knowledge about Pap test was 1.3 with a standard deviation of 0.36 (Table 2). The range of this score was 1–1.5. The participants’ level of knowledge about Pap test was between high to medium.

**Table 2:** Knowledge and intentions scales.

	Mean	*SD*
Knowledge	1.3	0.36
Intention	2.88	1.46

The dependent variable, intentions to uptake Pap smear, was measured using the Behavioral Intention Scale. The item response options included a 5-point Likert scale ranging from 1 (*likely*) to 5 (*unlikely*). On the Behavioral Intention Scale, a score of 2.5 was considered average. The data analysis revealed that the average score was 2.88 with a standard deviation of 1.46 (Table 2). The range of this scale score was 1–5. These findings indicated a consensus that the intentions to uptake Pap smear among the participants were above the average. In relation to the intentions to have Pap smears and how often participants intend to have the test, 56.5% (n = 264) of participants reported “Likely & Somewhat Likely”, followed by “Neutral” 15.6% (n = 73), and “Somewhat Unlikely” 14.3% (n = 67) to intend to have the test. Approximately 25.7% (n = 120) of participants reported “likely” to their intention to uptake the test yearly and about 38.9% (n = 182) reported “Somewhat Unlikely”.

Responses given by the participants in this study showed a positive correlation between knowledge about Pap test and intentions to uptake the test (r = 0.63) (Table 3). This correlation was not statistically significant (p > 0.05). This suggests that Saudi Arabian women with high knowledge about Pap test are more likely to have an intention to take the test. Responses given by the participants in this study showed a moderate negative correlation (r = -0.50, p > 0.05) between the knowledge about Pap test and age (Table 3). This indicates that younger participants tend to have higher levels of knowledge. The study revealed a significant negative correlation (r = -0.83, p<0.05) between age and intention (Table 3). This suggests that younger participants tend to have higher intention to perform Pap smear test. Also, the study found a significant positive correlation (r = 0.93, p<0.01) between marital status and intention (Table 3). Thus, the result of the study indicated that the demographic factors such as age and marital status are determinants of the intentions.

**Table 3:** Correlations between knowledge, intentions, age, and marital status.

	1	2	3	4
**1 Knowledge**	–	0.63*	-0.50*	
**2 Intention**		–	-0.83**	0.93**
**3 Age**			–	
**4 Marital status**				–

Data presented as Pearson correlation coefficient

** indicate significance in bold at p <0.01 and

* indicate non significance at > 0.05.

## Discussion

This study aimed to investigate Saudi Arabian women knowledge of Pap testing in relation to their intention to undergo the test. Majority of participant were aged 18–29 and more than half of the respondent were divorced. Most of the respondents were educated with a bachelor’s degree. The study found that average level of knowledge was 1.3 which is between high and moderate knowledge, and the average score for the intention was 2.88, to indicate that the intentions to uptake Pap smear among the participants were above the average. These findings suggest that study participants have levels of knowledge and intention to undergo Pap smear testing that are above average. Based on this study’s findings, the current level of knowledge among the participants do not concur with previous study indicating a low level of Pap screening awareness among Korean students [12].

The major findings of this study showed a positive correlation between knowledge about Pap test and intentions to uptake the test, but not statistically significant. It is possible that, due to social desirability bias, most of the participants reported having higher knowledge and intention to doing the test than they had. Also, the study revealed a significant correlation between demographic factors (age and marital status) and intention. The result showed that there was a significant relationship between marital status and the intention, which could be attributed to high percentages of married and divorced participants in the study, who have been sexually active to consider doing the test. This result concurs with findings reported by a previous study that the intention to undergo Pap testing differed by level of sexual experience [12]. Also, the results revealed a significant negative relationship between age and intention, suggesting that the intention of doing Pap smear decreased as people age.

However, the findings that there was no significant relationship between the knowledge of Pap smear test and age contradicted the literature that there was a positive relationship between demographic variables particularly ‘age’ and knowledge of Pap smear test [15, 16]. Overall, the findings of this study that no significant relationship was found between knowledge of Pap smear and the intention to perform Pap smear test means that knowledge cannot be a predictor to perform Pap smear test. This result is consistent with a previous study indicating that the level of awareness can not be related to the intention to adopt the preventive behavior [12].

## Conclusion

This study examined the knowledge of Pap smear in relation to the intention to undergo Pap testing. It found that study participants have levels of knowledge and intention to undergo Pap smear testing that are above average. The study revealed a significant correlation between demographic factors (age and marital status) and intention. This study offers new insights applicable to health institutions and health professional practice. Findings from the current study can inform health care providers about Saudi Arabian women knowledge of Pap smear and the intention to uptake the test. These findings can help health educators to design appropriate programs, awareness messages, and community campaigns to increase the knowledge and health beliefs about cervical cancer and its preventive behaviors. Even though participants’ levels of knowledge and their intentions to uptake Pap smear test were above average, these perceptions and intentions could be not reflected in adopting preventive behaviors. Therefore, there is a need to foster educational programs to increase the level of knowledge in women toward Pap smear test and influence their intention to adopt preventive behaviors. The results of this study indicate that continued research in this area is warranted. Future research could, for example, assess the knowledge and women’s perceptions related to barriers, facilitators, and resource information about the Pap smear test.

## Supporting information

S1 File Knowledge and intentions of the Pap smear survey. (DOCX)
