# Supplementary material for: Knowledge and intentions regarding the Pap smear test among Saudi Arabian women
Source: PLoS One. 2021 Jun 24;16(6):e0253850. doi: 10.1371/journal.pone.0253850 (PMC8224882; doi:10.1371/journal.pone.0253850)
Supplement: S1 File — (DOCX) [file pone.0253850.s001.docx]

**Knowledge and Intentions of the Pap Smear Survey**

| **Section 1: Demographic Questions** |
| --- |

1. What is your age in years? _______________

2. What is your marital status?

Single Married Separated/Divorced Widowed Other___________

3. What is the highest degree or level of school you have completed?

No schooling completed

Some high school, no diploma

High school graduate, diploma or the equivalent

Some college credit, no degree

Associate degree

Bachelor’s degree

Graduate degree

| **Section 2: Knowledge of the Pap Smear Scale** |
| --- |

1. Pap smears is most helpful way to detect pre-cancer and cancer of cervix

Yes

No

1. Women should have Pap smears at least every three years

Yes

No

1. Pap smear is not able to detect pre-cancerous cells before manifeastation of its symptoms

Yes

No

1. The purpose of the Pap smear is to detect abnormal cells in the cervix

Yes

No

1. Pap smear is not successful in reducing incidence and mortality of cervical cancer

Yes

No

1. Pap smear is able to detect all types of female genital cancer

Yes

No

1. Pap smear is a non-invasive and relatively inexpensive method

Yes

No

1. Women should have Pap smear since the onset of sexual activity

Yes

No

1. In Pap smear cervical cells are examined

Yes

No

1. Pap smears can be performed at both menstrual and non-menstrual period

Yes

No

1. A woman should not have sex 24 hours before having a Pap smear

Yes

No

1. Pap smears should be discontinued after menopause

Yes

No

1. If someone is having a normal Pap smear, she does not need Pap smears in the future

Yes

No

| Section 3: Behavioral Intention Scale  Please circle the number that represents your response to each question. |
| --- |

1. In the coming three months, I intend to have Pap smears.

1 2 3 4 5

likely Unlikely

2. I intend to have Pap smears yearly?

1 2 3 4 5

likely Unlikely

3. Next year , I am planning to having the Pap smears?

1 2 3 4 5

likely Unlikely

**استبيان لقياس المعرفة والنوايا تجاه مسحة عنق الرحم**

| **القسم 1: أسئلة ديموغرافية** |
| --- |

1-ما هو عمرك بالسنوات؟ _________

ما هي حالتك الاجتماعية؟-2

      _____ أعزب ___متزوج___ منفصل / مطلق___ أرمل____ آخر

3-ما هي أعلى درجة أو مستوى دراسي أكملته؟

                        لم يكتمل التعليم

                        بعض المدارس الثانوية ، لا يوجد دبلوم

                        خريج الثانوية العامة أو دبلوم أو ما يعادلها

            بعض الائتمان الجامعي ، بدون شهادة

           شهادة جامعية

           درجة بكالوريوس

           درجة التخرج

| **القسم 2 :: مقياس معرفة مسحة عنق الرحم** |
| --- |

1-مسحة عنق الرحم هي الطريقة الأكثر فائدة للكشف عن سرطان عنق الرحم وسرطانه

نعم

لا

2-يجب أن تخضع المرأة لفحص عنق الرحم كل ثلاث سنوات على الأقل

نعم

لا

 مسحة المهبل ليست قادرة على اكتشاف الخلايا قبل السرطانية قبل   3-

نعم

لا

 الغرض من مسحة عنق الرحم هو الكشف عن الخلايا غير الطبيعية في عنق الرحم  4-

نعم

لا

 لم تنجح مسحة عنق الرحم في الحد من الإصابة بسرطان عنق الرحم والوفيات  5-

نعم

لا

6- مسحة عنق الرحم قادرة على الكشف عن جميع أنواع سرطان الأعضاء التناسلية الأنثوية  6-

نعم

لا

مسحة عنق الرحم هي طريقة غير جراحية وغير مكلفة نسبيًا     7-

نعم

لا

يجب أن تخضع النساء لمسحة عنق الرحم منذ بداية النشاط الجنسي     8-

نعم

لا

في مسحة عنق الرحم يتم فحص خلايا عنق الرحم   9-

نعم

لا

يمكن إجراء مسحة عنق الرحم في كل من الدورة الشهرية وغير الشهريه-10

نعم

لا

يجب ألا تمارس المرأة الجنس قبل 24 ساعة من إجراء مسحة عنق الرحم -11

نعم

لا

يجب إيقاف مسحة عنق الرحم بعد سن اليأس -12

نعم

لا

إذا كان لدى شخص ما مسحة عنق الرحم العادية ، فلن تحتاج إلى مسحة عنق الرحم في المستقبل -13

نعم

لا

| **القسم :3مقياس النية السلوكية**  **يرجى وضع دائرة حول الرقم الذي يمثل إجابتك على كل سؤال** |
| --- |

في الأشهر الثلاثة القادمة ، أنوي إجراء مسحة عنق الرحم.1

                        1 2 3 4 5

محتمل غير محتمل

2.أنوي إجراء مسحة عنق الرحم سنويًا؟

                        1 2 3 4 5

محتمل غير محتمل

.في العام القام العام ، أخطط لإجراء مسحة عنق الرحم؟3

                        1 2 3 4 5

محتمل غير محتمل
